# Medication Dosage Impact on Mortality in Old-Age Individuals with Schizophrenia: A National Cohort Study

**DOI:** 10.3390/ph17010078

**Published:** 2024-01-08

**Authors:** Jia-Ru Li, Ling-Ling Yeh, Ji-Yu Lin, Yi-Ju Pan

**Affiliations:** 1 Department of Psychiatry, Far Eastern Memorial Hospital, New Taipei City 22060, Taiwan; 2 Institute of Epidemiology and Preventive Medicine, College of Public Health, National Taiwan University, Taipei 10025, Taiwan; 3 Graduate School of Humanities and Social Sciences, Dharma Drum Institute of Liberal Arts, New Taipei City 20842, Taiwan; yehll@dila.edu.tw; 4 Department of Psychiatry, National Taiwan University Hospital Yunlin Branch, Yunlin 64041, Taiwan; b98401115@ntu.edu.tw; 5 Department of Chemical Engineering and Materials Science, Yuan Ze University, Taoyuan City 32003, Taiwan

**Keywords:** schizophrenia, old-age, antipsychotic, mood stabilizer, antidepressant, sedative-hypnotic, polypharmacy, daily defined dosage, mortality

## Abstract

As the prevalence of old-age individuals with schizophrenia (OAS) increases in a society undergoing demographic aging, the exploration of medication choices becomes increasingly crucial. Due to the current scarcity of literature on OAS, this study seeks to examine how the utilization and cumulative dosages of psychotropic medications influence both overall and cause-specific mortality risks within this population. A national cohort of 6433 individuals diagnosed with OAS was followed up for 5 years. This study involved comparing the mortality rates associated with low, moderate, and high dosages of antipsychotics, antidepressants, mood stabilizers, and sedative/hypnotic drugs against the ‘no exposure’ category, based on individual dosages. Cox regression was employed for survival analyses to compare overall mortality and specific-cause mortality across various dosage groups. The exposure variable examined was the dosage of a specific psychotropic medication. Covariates were adjusted accordingly. The analysis revealed that patients on low/moderate antipsychotic doses had improved survival compared to non-exposed individuals. Moderate antipsychotic use corresponded to reduced cardiovascular disease mortality risk. Similarly, those exposed to antidepressants had enhanced survival in low and moderate doses. Sedative-hypnotic exposure was linked to decreased mortality risk in low doses. This study observed that low/moderate antipsychotic doses in older adults with schizophrenia were associated with decreased all-cause mortality, emphasizing the significance of precise medication selection and dosing. It underscores the need for vigilant polypharmacy management and tailored medication strategies in addressing the complexities of treating OAS.

## 1. Introduction

The aging of the global population stands as an indisputable and ongoing phenomenon, shaping the demographic landscape worldwide. With 2021 statistics revealing that roughly one in every ten individuals globally had reached the age of 65 or above [1], the significant impact of this demographic shift has been highlighted. Schizophrenia has a prevalence among older adults of approximately 0.1–0.5% compared to the lifetime prevalence of schizophrenia at about 1% [2]. Old-age individuals with schizophrenia (OAS) can be divided into two major groups: new onset schizophrenia patients in later life (onset from 40 to 60 years old, late-onset schizophrenia, LOS; onset later than 60, very-late-onset schizophrenia-like psychosis, VLOSLP) [3], and those with onset from an early age who have grown old, called older people with chronic schizophrenia. Both groups generally lack comprehensive research to find better treatment guidelines. While some OAS may undergo an age-related improvement in positive symptoms, a predominant number continue to grapple with negative symptoms, cognitive deficits, depression, enduring side effects resulting from prolonged antipsychotic usage, and concurrent medical conditions [4]. Consequently, OAS could potentially emerge as a noteworthy public health concern in an aging society.

Pharmacological treatments are available for management of OAS but may be limited by tolerability, presence of physical comorbidities, and potential adverse effects of polypharmacy [5,6]. A more recent study has indicated that patients with old-age onset schizophrenia share a unique clinical course and treatment response compared to the general schizophrenia population [7,8]. Considering that OAS may have physical comorbidities and individuals may be maintained on psychotropic medications long term, the accumulative dosage and longitudinal follow-up for its impact on health outcomes or mortality is of clinical significance [9]. However, to date, there has been no research specifically focusing on the associations between exposure dosage of different psychotropic medications and mortality in OAS. In previous literature, the exploration of medication dosage involved grouping mainly based on the comparison of patients with any antipsychotic use versus no use or the100-mg chlorpromazine equivalents as a differentiation criterion [10,11]. With regards to adult patients with schizophrenia, a Swedish study has reported that moderate and high-dose antipsychotic and antidepressant use were associated with lower overall mortality and that high-dose benzodiazepine was associated with elevated mortality risk, in comparison with no exposure [12]. In Asia, one previous study investigating the mortality risk and the impact of various classes of psychotropic medications in individuals with schizophrenia aged over 15 years [13] found that adequate dosages of antipsychotics and antidepressants are associated with lower mortality risks.

In an aging society, exploring the medication choices for OAS and understanding their implications on health outcomes stands as a pivotal and imperative research area. Given the current lack of sufficient literature on OAS, this study aims to investigate the impact of psychotropic medication usage and cumulative dosages on the risk of all-cause and cause-specific mortality in this geriatric population. Additionally, recognizing the potential impacts of polypharmacy in OAS as an important issue, this study analyzes the relationship between medication and the risk of mortality, considering both the types and dosages of concurrently administered drugs, including antipsychotics, antidepressants, mood stabilizers, and sedative-hypnotics, using a national cohort of elderly patients with schizophrenia from Taiwan’s national database for healthcare services and linked to the national mortality registry to identify cause of death.

## 2. Results

The present study comprised 6433 patients diagnosed with OAS. Table 1 provides a summary of the demographic and clinical traits observed within this cohort, illustrating that among all the included OAS, 59.9% (*n* = 3843) were females. The mean age was 73.23 ± 6.6 years. Among the OAS patients, 62.9% (*n* = 4049) had received a catastrophic illness certification. This certification provides the main benefit of reducing patients’ financial burden by covering a portion of their medical expenses and relieving some of the burden associated with health insurance. Additionally, 14.7% (*n* = 948) of OAS came from lower-income households. Approximately 13.3% (*n* = 853) of OAS patients had experienced hospitalization in psychiatric wards during the initial year following diagnosis, suggesting a heightened severity level for these OAS patients. During the five-year follow-up, 31.9% (*n* = 2053) of OAS patients died (Table 1). Of all those who died during the follow-up, 97.0% (*n* = 1992) died of natural causes; only 3.0% (*n* = 61) died an unnatural death, including suicides (*n* = 15) and other accidental deaths. Among those who died of natural causes, 13.0% (*n* = 258) died of cancer; 19.1% (*n* = 384) died of CVD; and 5.6% (*n* = 111) died due to DM-related causes.

**Figure 1:**
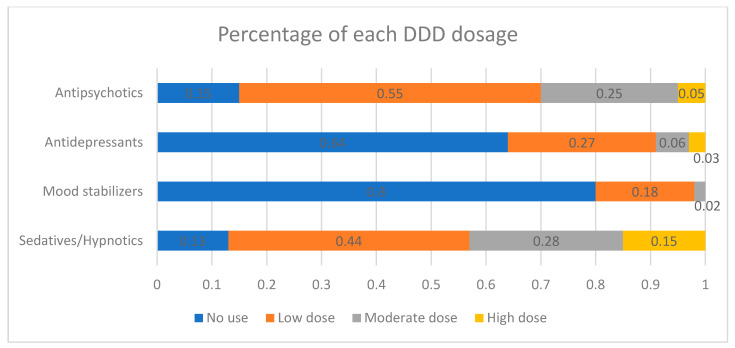
Percentage of each defined daily dose (DDD) dosage exposure of antipsychotics, antidepressants, mood stabilizers, and sedatives/hypnotics.

Figure 1 provides a summary of the percentage of each psychotropic agent within every DDD group. Among the entire OAS population, 14.7% (*n* = 944) had no exposure to antipsychotics, signifying that these patients did not receive treatment during the follow-up period.; 54.7% (*n* = 3520) had low exposure; 25.4% (*n* = 1636) had moderate exposure; and 5.2% (*n* = 333) had high exposure. With regards to mood stabilizers use, 79.8% (*n* = 5131) had no exposure. Approximately 36% of the OAS patients had exposure to antidepressants; 87% of the OAS patients had exposure to sedative-hypnotics (Figure 1).

Low and moderate exposure to antipsychotics was linked to lower risks of overall mortality when compared to the group without any exposure (hazard ratio (HR), with 95% confidence interval (CI): 0.77, 0.69–0.90; 0.73, 0.63–0.85, respectively). Moreover, moderate exposure to antipsychotics correlated with reduced risks of CVD mortality (HR, 95% CI: 0.62, 0.43–0.88). Similarly, both low and moderate exposure to antidepressants showed decreased risks of overall mortality (HR, 95% CI: 0.71, 0.64–0.80; 0.80, 0.66–0.97, respectively) and low exposure to antidepressants was associated with decreased risks of CVD mortality (HR, 95% CI: 0.59, 0.45–0.77). Compared to the no exposure group, low exposure to sedative-hypnotics was linked to reduced risks of both overall mortality and CVD mortality (HR, 95% CI: 0.86, 0.75–0.98; 0.66, 0.49–0.89) (Table 2).

**Figure 2:**
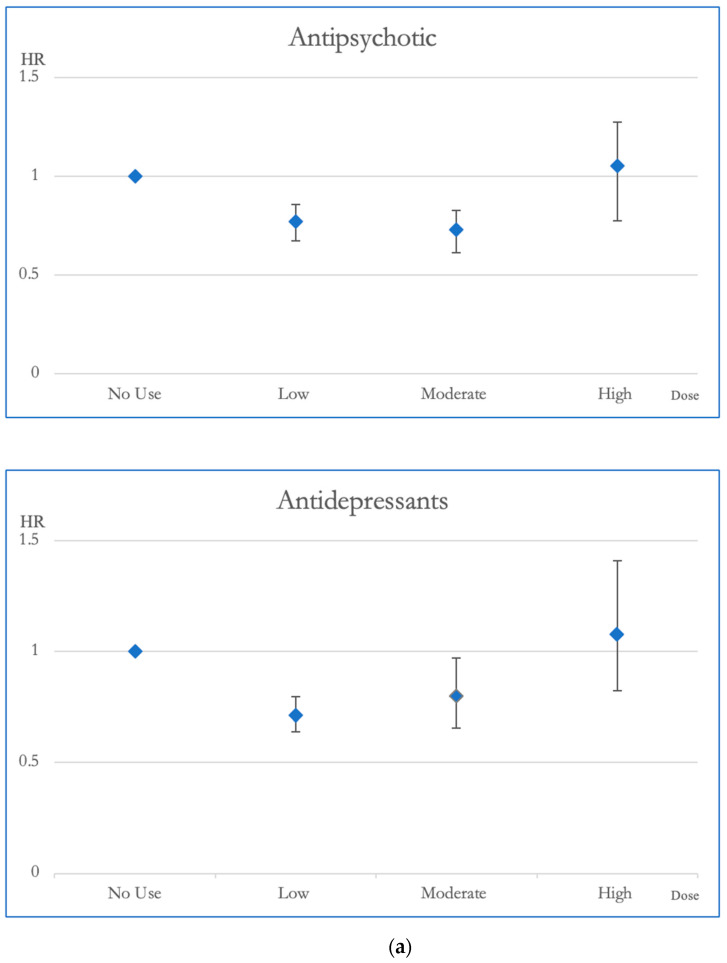

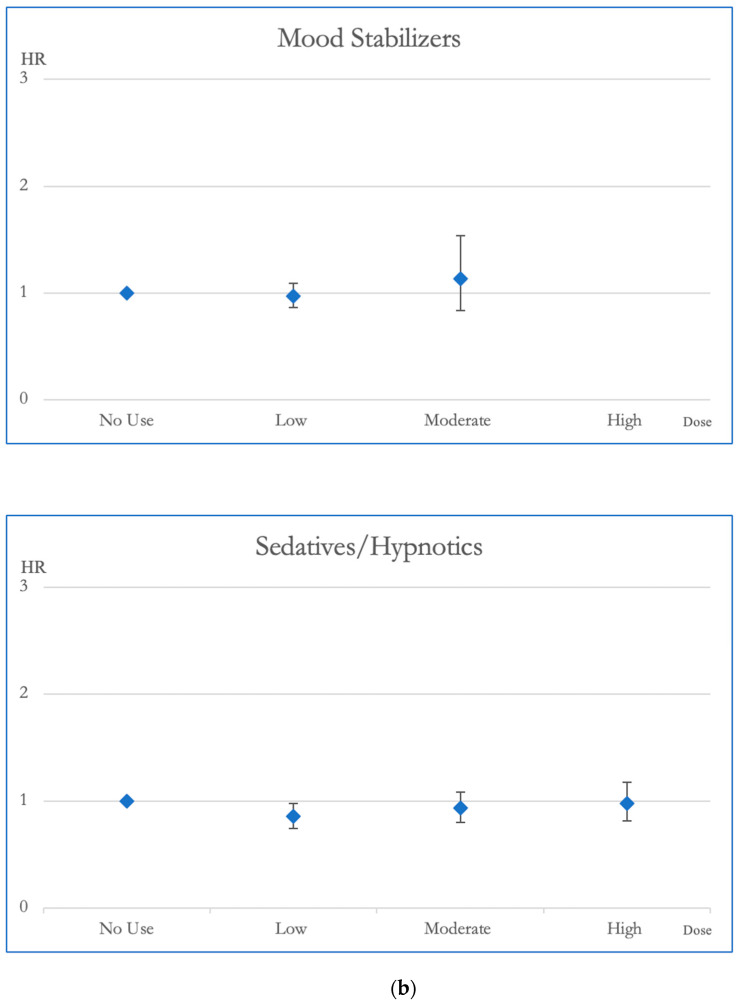
(**a**). Hazard ratios and 95% Confidence Intervals for the different exposures to antipsychotics and antidepressants for overall mortality. Survival analysis utilized Cox regressions and controlled for multiple variables: gender, age, socioeconomic status (insurance level, household income, and urbanization level), health condition (non-psychiatric health cost), disease severity (catastrophic illness card, psychiatric ward admission during the first year, and psychiatric-care-related cost), and concomitant psychotropic agent use. The hazard ratio for overall mortality was calculated across varying degrees of exposure for antipsychotics and antidepressants, which were categorized into four groups with no exposure, low exposure (<0.5 DDD), moderate exposure (0.5–1.5 DDD), and high exposure (>1.5 DDD). The scale of the vertical axis was adjusted by the level of the hazard ratios. (**b**). Hazard ratios and 95% Confidence Intervals for the different exposures to mood stabilizers and sedatives/hypnotics for overall mortality. Survival analysis utilized Cox regressions and controlled for multiple variables: gender, age, socioeconomic status (insurance level, household income, and urbanization level), health condition (non-psychiatric health cost), disease severity (catastrophic illness card, psychiatric ward admission during the first year, and psychiatric-care-related cost), and concomitant psychotropic agent use. The hazard ratio for overall mortality was calculated across varying degrees of exposure for mood stabilizers and sedatives/hypnotics, which were categorized into four groups with no exposure, low exposure (<0.5 DDD), moderate exposure (0.5–1.5 DDD), and high exposure (>1.5 DDD). The scale of the vertical axis was adjusted by the level of the hazard ratios.

**Figure 3:**
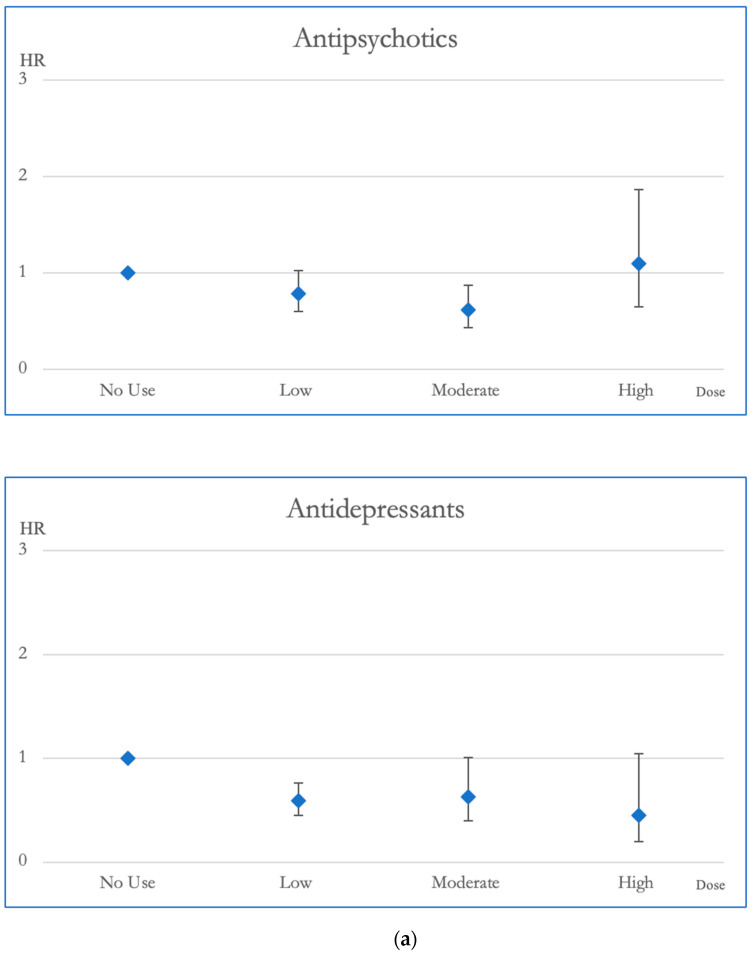

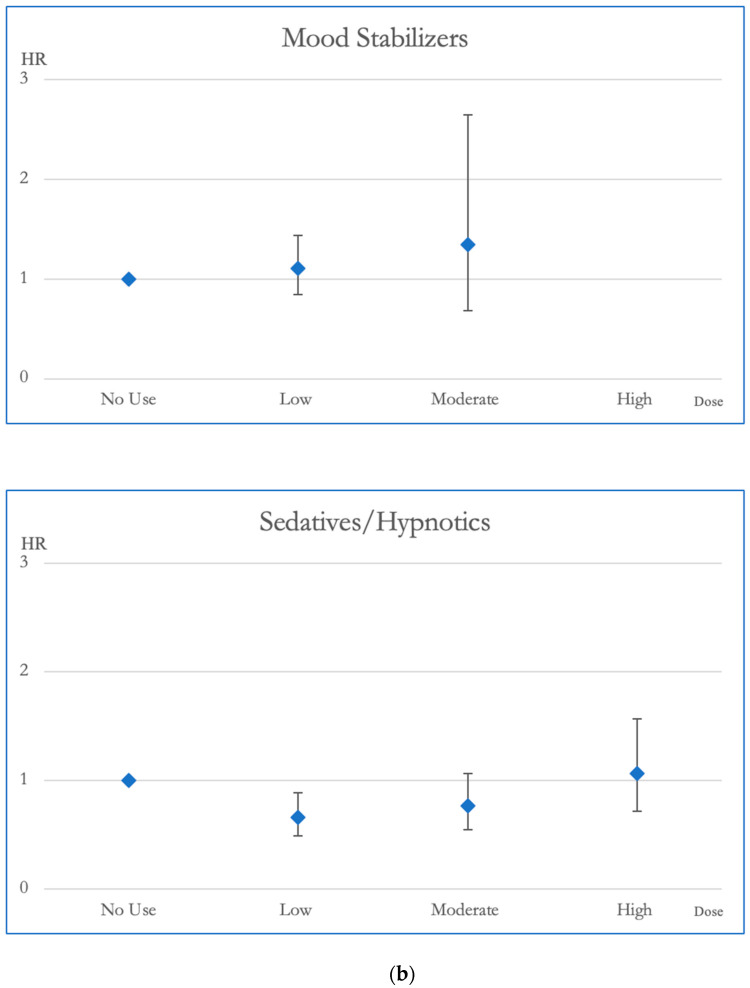
(**a**). Hazard ratios and 95% Confidence Intervals for the different exposures to antipsychotics and antidepressants for CVD mortality. Survival analysis utilized Cox regressions and controlled for multiple variables: gender, age, socioeconomic status (insurance level, household income, and urbanization level), health condition (non-psychiatric health cost), disease severity (catastrophic illness card, psychiatric ward admission during the first year, and psychiatric-care-related cost), and concomitant psychotropic agent use. The hazard ratio for overall mortality was calculated across varying degrees of exposure for antipsychotics and antidepressants, which were categorized into four groups with no exposure, low exposure (<0.5 DDD), moderate exposure (0.5–1.5 DDD), and high exposure (>1.5 DDD). The scale of the vertical axis was adjusted by the level of the hazard ratios. (**b**). Hazard ratios and 95% Confidence Intervals for the different exposures to mood stabilizers and sedatives/hypnotics for CVD mortality. Survival analysis utilized Cox regressions and controlled for multiple variables: gender, age, socioeconomic status (insurance level, household income, and urbanization level), health condition (non-psychiatric health cost), disease severity (catastrophic illness card, psychiatric ward admission during the first year, and psychiatric-care-related cost), and concomitant psychotropic agent use. The hazard ratio for overall mortality was calculated across varying degrees of exposure for mood stabilizers and sedatives/hypnotics, which were categorized into four groups with no exposure, low exposure (<0.5 DDD), moderate exposure (0.5–1.5 DDD), and high exposure (>1.5 DDD). The scale of the vertical axis was adjusted by the level of the hazard ratios.

Figure 2 and Figure 3 illustrate the cumulative impact of each category of psychotropic agent on the risk of all-cause mortality and CVD mortality in OAS patients. Compared to OAS individuals with no exposure to antipsychotics, those exposed to low and moderate dosages exhibited better survival outcomes in all-cause mortality. Mood stabilizer exposure did not show any discernible association with changes in all-cause mortality risk. Similarly, among OAS individuals, exposure to antidepressants at low and moderate dosages demonstrated improved survival outcomes in all-cause mortality compared to those without exposure. At lower doses, the use of sedative-hypnotic medications showed a correlation with a reduction in overall mortality (Figure 2).

In terms of CVD mortality, moderate dosages of antipsychotic exposure were linked to decreased risk compared to no exposure, demonstrating a reduction in HR of 0.617. Mood stabilizer exposure did not appear to be associated with changes in CVD mortality risk. Antidepressant medications, especially at low doses, displayed a link to decreased CVD mortality, highlighted by an HR of 0.59. Additionally, individuals exposed to sedative-hypnotics at low dosages exhibited better survival outcomes in CVD mortality compared to those without exposure, with an HR of 0.66 (Figure 3).

## 3. Discussions

The present study represents an initial exploration examining the correlations between the cumulative exposure to various psychotropic medications and the mortality risks in OAS. This investigation takes into account proxies of disease severity and dosages of concurrent medications (antipsychotics, antidepressants, mood stabilizers, and sedative-hypnotics). The findings suggest that in comparison to OAS patients who have no exposure to antipsychotics, those patients exposed to low and moderate doses showed decreased overall mortality. Similarly, both low and moderate exposure to antidepressants were associated with decreased risks of overall mortality. Low exposure to sedative-hypnotics was linked to reduced risks of overall mortality. Additionally, compared to OAS patients with no exposure, moderate exposure to antipsychotics, low exposure to antidepressants, and low exposure to sedative-hypnotics were associated with reduced CVD mortality. There does not seem to be a clear association between exposure dosage to mood stabilizers and changes in the risks of all-cause mortality or CVD mortality. These analyses highlight the intricate and significant relationship between medication exposure and dosage among OAS and mortality risks, further emphasizing the importance of adequate medication dosage in clinical considerations, particularly for older patients with schizophrenia.

There has been no existing literature on the exposure levels of psychotropic medications and mortality in OAS. Previous studies have indicated that exposure to antipsychotics led to improved survival outcomes in patients with schizophrenia [12,13], which aligns with the findings of the current study that OAS patients exposed to low and moderate doses of antipsychotics had a reduced risk of all-cause mortality. In recent years, atypical antipsychotics have been widely prescribed to elderly patients with psychotic symptoms due to their novel receptor binding profiles, effectiveness in addressing negative symptoms, and reduced extrapyramidal symptoms. Nevertheless, a higher incidence of adverse effects, including CVD, has been observed in elderly patients across various psychiatric disorders, including but not limited to schizophrenia, dementia, and mood disorders [14]. Furthermore, VLOSLP demonstrates higher rates of morbidity and mortality compared with schizophrenia among younger adults, which may be due to increased physical comorbidities and accidents in this geriatric group [15]. Despite both the increase in the prescriptions and concerns regarding the potential adverse effects, there have been very few discussions specifically concerning the mortality risk when using antipsychotics in OAS patients. In the present study, we found that OAS patients have decreased all-cause mortality with antipsychotic treatment in low and moderate doses, compared to those with no antipsychotic usage. These conclusions are drawn from data collected in actual clinical settings in Taiwan. Although additional research is required to verify this finding in the future, employing adequate low to moderate dosages and individualizing dose adjustments based on the specific physical conditions of OAS would be a more suitable strategy for clinical intervention.

Furthermore, we found that antipsychotic exposure seems to be related to decreased CVD mortality in moderate dosages compared to no exposure with an HR of 0.617. In line with our findings of OAS, a Swedish national database study demonstrated that the utilization of antipsychotics at low and moderate doses correlated with a reduction in CVD mortality among adult schizophrenia patients [12]. Torniainen et al. proposed a U-shaped mortality curve with regards to dose range to explain the excessive CVD mortality in adult schizophrenia patients [16]. One Korean cohort study found that the use of antipsychotic medication reduced the risk of death from ischemic heart disease and stroke. However, there was no observed impact on the risk of death from nonischemic heart disease [17]. Additionally, the utilization of antipsychotic drugs contributed to the improvement of psychiatric symptoms in individuals with schizophrenia. This improvement could potentially enhance their capacity to seek medical assistance, compliance with treatment, and enhance their self-care practices. These factors may also collectively contribute to a reduced risk of mortality, including CVD [18]. Considering that the present study found that moderate exposure to antipsychotic drugs had the lowest HR for both overall mortality and CVD mortality, prescribing antipsychotics within an appropriate dosage range may be linked to reduced mortality risks in OAS.

Our research findings indicate that among OAS, the use of antidepressant medication at low and moderate doses is linked to a reduction in overall mortality. Additionally, across all dose levels, antidepressant exposure is linked to a reduction in CVD mortality in this geriatric population. Combining antipsychotics with antidepressants is a frequently used clinical approach to address symptoms in individuals with schizophrenia [19]. Tiihonen et al. found a decline in all-cause mortality among schizophrenia patients receiving antidepressant treatment compared to those without exposure to antidepressants, aligning with our own findings [12]. There is some evidence suggesting an effect of combining an antidepressant with antipsychotic treatments in improving depression and the negative symptoms of schizophrenia [20,21]. While adjunctive antidepressant prescription for schizophrenia is not common in Asia [22], clinicians may find it rational to combine antidepressants with antipsychotics for managing negative symptoms or depressive symptoms [23]. Previous studies have reported an increased risk of adverse outcomes, such as all-cause mortality, myocardial infarction, and stroke, associated with antidepressant use in older adults with depression [24]. However, some studies have proposed that antidepressants may have cardiovascular protective effects. These effects include cardioprotective actions through inhibiting platelet activation, reducing inflammation, improving endothelial function, and regulating cardiac function [25,26,27,28]. Antidepressants may also have cardio-protective effects on ventricular function and the cardiac conduction system [27]. It is also worth noting that dysfunction of the hypothalamic–pituitary–adrenal (HPA) axis may be one of the biomarkers of schizophrenia [29]. The observed effect of reduced CVD mortality in OAS using antidepressants may be linked to the regulation of the HPA axis in this geriatric population [30]. While these findings underscore the potential role of antidepressant medication in OAS, there is a simultaneous need for a deeper exploration of the effects and potential risks associated with these drugs.

The current study demonstrated that the use of sedative-hypnotics at a low dosage is linked to a substantial decrease in both all-cause and CVD mortality among individuals diagnosed with OAS, displaying HRs of 0.855 and 0.685, respectively. In the treatment of schizophrenia, benzodiazepines are commonly prescribed alongside antipsychotic medications to alleviate symptoms of anxiety, sleep disorders, agitation, and antipsychotic-related side effects [31,32,33]. Approximately 20% of OAS were prescribed benzodiazepines in Asia [34]. In Taiwan, there seems to be prolonged sedative-hypnotics use among OAS patients [35]. The earlier study conducted in Taiwan indicated that exposure to sedative-hypnotics was linked to a slight increase in overall mortality among individuals aged 15 or above with schizophrenia [13]. Furthermore, the use of sedative-hypnotics in the elderly population may increase the risk of falls and dementia as well as elevate the risk of polypharmacy and drug interactions [36,37,38]. However, a cohort study in the Netherlands revealed that benzodiazepines did not have a significant impact on mortality rates among individuals with schizophrenia [39]. Another Swedish national cohort study found that persistent, high-dose utilization of benzodiazepines among individuals diagnosed with schizophrenia was linked to increased rates of both overall mortality and CVD mortality, whereas low-dose benzodiazepine use did not affect mortality rates [12]. Therefore, the correlation between the use of sedative-hypnotics and the mortality risk in schizophrenia patients may differ by dosage. While the precise underlying mechanisms remain elusive, the utilization of low-dose sedative-hypnotics among OAS patients does not conclusively demonstrate harm; rather, it potentially presents a nuanced equilibrium between benefits and drawbacks based on existing research. The urgent pursuit of rigorous investigations is imperative to elucidate the precise impact of sedative-hypnotic use in OAS. Therefore, the prescription of sedative-hypnotics in this geriatric cohort demands meticulous consideration and an in-depth assessment of its potential advantages and disadvantages.

The strengths of the current study include national coverage and encompassing OAS patients in all clinical settings. In addition, this study had a 5-year consecutive follow-up period and calculated the DDDs for each category of psychotropic medications, providing rarely available information regarding the type and degree of medication exposure in this geriatric population. Furthermore, the link with the national mortality registry provided information regarding the causes of death. However, this study had several limitations. First, it was a non-randomized study design, and we needed to be cautious when interpreting the results due to potential selection bias. Furthermore, we did not adjust for comorbidities, such as hepatic and renal failure, which might have affected mortality. Additionally, the lack of accurate information on disease severity and patient lifestyles, such as alcohol use and smoking habits, in the NHIRD restricted the assessment of these factors. 

## 4. Materials and Methods

National Health Insurance in Taiwan is a single-payer, compulsory social insurance system, which provided coverage to a total of 23.8 million people in 2018, with a coverage rate of 100% [40]. Taiwan’s National Health Insurance Research Database (NHIRD) was established for scientific and study purposes. The NHIRD contains the characterizing data of insured residents, including basic demographic characteristics, expenditures, medical procedures, and medications. The *International Classification of Diseases*, *Ninth Revision*, *Clinical Modification* (ICD-9-CM) was used for diagnosis in the NHIRD before 2016 [41].

**Figure 4:**
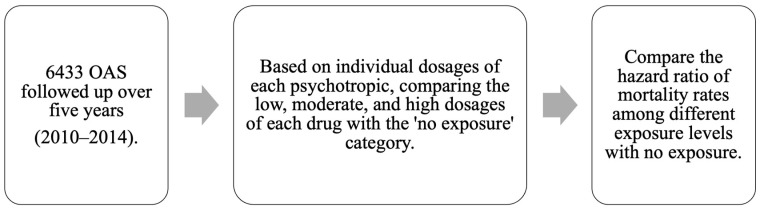
Flow chart of the study design. OAS, old-age individuals with schizophrenia.

The present study was approved by the Research Ethics Review Committee of Far Eastern Memorial Hospital in Taiwan (109150-E). Individuals aged ≥ 65 years and diagnosed with schizophrenia (ICD-9-CM category 295) by psychiatrists in 2010 were identified from Taiwan’s NHIRD, which was provided by the Health and Welfare Data Science Center of the Ministry of Health and Welfare in Taiwan, and followed up for the consecutive five years (2010–2014). The index date refers to the specific date when the person received their initial diagnosis of schizophrenia in 2010. Connections between mortality results and causes of death were established using Taiwan’s national mortality registry. Information regarding age, gender, socioeconomic factors (such as household income, insurance premium, and urbanization level), and whether the person obtained a catastrophic illness certification—issued by the National Health Insurance Administration to assist patients coping with significantly impactful diseases—was collected from the data on the index date (Figure 4).

The mean defined daily dose (DDD) refers to the assumed average daily dose for maintenance for its main indication in adults; referenced with the guidelines for DDD by the World Health Organization [42]. We determined the mean DDD for antipsychotics, antidepressants, mood stabilizers, and sedative-hypnotics individually. This was achieved by dividing the total doses by the duration of follow-up days. Following this, we classified each medication into four categories: no exposure, low exposure (<0.5 DDD), moderate exposure (0.5–1.5 DDD), and high exposure (>1.5 DDD).

For descriptive analyses, we first compared the demographic and socioeconomic characteristics across OAS patients of different exposure groups. Categorical variables were analyzed using the chi-squared test and continuous variables were analyzed using F tests. We utilized Cox regression for survival analyses to compare overall mortality and specific-cause mortality across different dosage groups. The exposure variable was the exposure dosage of a psychotropic medication of interest. The factors considered as covariates encompassed age, gender, socioeconomic status (such as insurance premium level, household income, and urbanization level), healthcare expenses unrelated to psychiatric care in the initial year post-diagnosis serving as an indicator for general medical conditions, proxies for disease severity (including holding a catastrophic illness certification and admission to psychiatric wards during the first year of diagnosis), and concomitant use of psychotropic medication dosages. The statistical significance was set at a *p* value of 0.05. All statistical analyses were performed using SPSS (version 21.0; IBM, Armonk, NY, USA). 

## 5. Conclusions

In summary, our findings indicate that OAS individuals who were exposed to low or moderate doses of antipsychotics had a lower risk of all-cause mortality compared to those with no antipsychotic exposure. Moderate exposure to antipsychotic medications was associated with the lowest HR for CVD mortality. This suggests that prescribing antipsychotics within an appropriate dosage range may be linked to reduced mortality risks in OAS. Among OAS, the present study showed that the use of antidepressants in low and moderate doses was associated with reduced all-cause mortality compared to those without antidepressant exposure, and low exposure to antidepressants showed the lowest HR for CVD mortality. Furthermore, low exposure to sedative-hypnotics was linked to decreased overall mortality and CVD mortality. Polypharmacy in older adults demands extra vigilance, necessitating not only careful medication selection but also meticulous dosage adjustments, taking into account the individual’s physical comorbidities and the potential drug–drug interactions from other diseases.

## Figures and Tables

**Table 1:** Demographic and clinical characteristics of old age schizophrenia patients (*n* = 6433) by different antipsychotic exposure.

	Total (*n* = 6433)	No Antipsychotic Exposure (*n* = 944, 14.7%)	Low Antipsychotic Exposure (*n* = 3520, 54.7%)	Moderate Antipsychotic Exposure (*n* = 1636, 25.4%)	High Antipsychotic Exposure (*n* = 333, 5.2%)	Significance
Age (years old) [mean (SD)]	73.23 (6.6)	74.6 (7.0)	74.6 (7.0)	71.5 (5.4)	70.0 (4.2)	*F* = 126.75
Gender [*n* (%)]						*χ*² = 10.049 *
Female	3843 (59.9)	531 (56.3)	2117 (60.1)	976 (59.7)	219 (65.7)	
Male	2590 (40.1)	413 (43.7)	1403 (39.9)	660 (40.3)	114 (34.3)	
Lower-income household [*n* (%)]	948 (14.7)	141 (14.9)	473 (13.4)	288 (17.6)	46 (13.8)	*χ*² = 15.688
With catastrophic illness card ^a^ [*n* (%)]	4049 (62.9)	559 (59.2)	2012 (57.2)	1217 (74.4)	261 (78.4)	*χ*² = 182.005
Psychiatric healthcare cost [mean (SD)]	45,228 (88,433)	11,856 (38,399)	32,440 (70,199)	79,285 (112,981)	107,770 (133,018)	*F* = 227.33 *
Non-psychiatric healthcare cost ^b^ [mean (SD)]	79,421 (161,005)	100,910 (208,438)	87,048 (169,539)	53,863 (99,821)	63,449 (140,242)	*F* = 23.31 *
Psychiatric ward admission ^c^ in the 1st year [*n* (%)]	853 (13.3)	35 (3.7)	387 (11.0)	345 (21.1)	86 (25.8)	*χ*² = 223.482
Death [*n* (%)]						
All causes	2053 (31.9)	379 (40.1)	1179 (33.5)	396 (24.2)	99 (29.7)	*χ*² = 78.974
Natural causes	1992 (31.0)	368 (39.0)	1154 (32.8)	377 (23.0)	93 (27.9)	*χ*² = 687.98 *
Cancer	258 (4.0)	51 (5.4)	139 (3.9)	53 (3.2)	15 (4.5)	
CVD	384 (6.0)	73 (7.7)	227 (6.4)	64 (3.9)	20 (6.0)	
DM	111 (1.7)	12 (1.3)	72 (2.0)	23 (1.4)	4 (1.2)	
Unnatural causes	61 (0.9)	11 (1.2)	25 (0.7)	19 (1.2)	6 (1.8)	*χ*² = 45.19 *
Suicide	15 (0.2)	1 (0.1)	9 (0.3)	4 (0.2)	1 (0.3)	
Unknown	13 (0.2)	3 (0.3)	3 (0.1)	7 (0.4)	0 (0.0)	
Follow-up days [mean (SD)]	1585.69 (456.0)	1350.44 (635.2)	1508.32 (500.5)	1580.90 (465.3)	1524.81 (515.6)	*F* = 40.42

Continuous variables were compared using ANOVA; categorical variables were compared via chi-squared test. ^a^ People diagnosed by a physician as having a condition classified as a catastrophic illness by the Ministry of Health and Welfare can apply for a catastrophic card with which they do not need to pay a co-payment for obtaining care for the illness. ^b^ The non-psychiatric healthcare cost during the first year after diagnosis served as a proxy for the patient’s general physical health condition. ^c^ Admission to a psychiatric ward during the first year after diagnosis served as a proxy for the severity of a patient’s psychiatric illness. SD = standard deviation; CVD = cardiovascular disease; DM = diabetes mellitus. * *p* < 0.001.

**Table 2:** Adjusted hazard ratios for antipsychotics, mood stabilizers, antidepressants, and sedative-hypnotics by DDD group based on degree of exposure in individuals with old age schizophrenia.

	Low Exposure		Moderate Exposure		High Exposure	
	Adjusted Hazard Ratio	95% CI	Adjusted Hazard Ratio	95% CI	Adjusted Hazard Ratio	95% CI
**Overall mortality**						
Antipsychotics	0.773 **	0.687–0.896	0.729 **	0.629–0.846	1.054	0.834–1.333
Mood stabilizers	0.973	0.866–1.093	1.134	0.835–1.539		
Antidepressants	0.714 **	0.640–0.797	0.797 *	0.656–0.970	1.077	0.823–1.410
Sedative-Hypnotics	0.855 *	0.745–0.982	0.934	0.803–1.086	0.979	0.816–1.175
**Cardiovascular mortality**						
Antipsychotics	0.788	0.603–1.031	0.617 **	0.434–0.878	1.102	0.651–1.867
Mood stabilizers	1.103	0.845–1.440	1.342	0.681–2.647		
Antidepressants	0.589 **	0.453–0.766	0.632	0.396–1.011	0.454	0.198–1.043
Sedative-Hypnotics	0.658 *	0.489–0.886	0.763	0.548–1.063	1.062	0.719–1.567
**Cancer mortality**						
Antipsychotics	0.688 *	0.497–0.954	0.660 *	0.440–0.989	1.076	0.585–1.982
Mood stabilizers	0.877	0.626–1.230	0.901	0.366–2.221		
Antidepressants	0.827	0.607–1.126	1.170	0.700–1.956	2.194 *	1.174–4.100
Sedative-Hypnotics	0.762	0.526–1.104	0.755	0.502–1.135	0.507 *	0.297–0.864
**DM mortality**						
Antipsychotics	1.435	0.776–2.655	0.959	0.466–1.973	0.783	0.243–2.528
Mood stabilizers	0.882	0.531–1.464	0.804	0.194–3.336		
Antidepressants	0.599 *	0.378–0.949	0.356 *	0.127–0.994	0.363	0.086–1.535
Sedative-Hypnotics	1.408	0.683–2.900	1.842	0.869–3.905	2.866 *	1.265–6.492

Survival analysis utilized Cox regressions and controlled for multiple variables: gender, age, socioeconomic status (insurance premium level, lower-income household, and urbanization level), proxy for general physical health condition (non-psychiatric healthcare cost), proxy for disease severity (catastrophic illness card and psychiatric ward admission during the first year), and concomitant psychotropic agent use. Hazard ratios for overall mortality, cardiovascular mortality, and suicide mortality among old-age individuals with schizophrenia were calculated by DDD group based on degree of exposure for antipsychotics, mood stabilizers, antidepressants, and sedative-hypnotics. Patients were categorized into four DDD groups: no exposure (the reference group), low exposure (<0.5 DDD), moderate exposure (0.5–1.5 DDD) and high exposure (>1.5 DDD). CI = confidence interval. * *p* < 0.05, ** *p* < 0.001.

## Data Availability

Data is contained within the article.
